# Bilirubin in Parkinson’s disease: pathogenic mechanisms and therapeutic potentials

**DOI:** 10.3389/fnins.2026.1806875

**Published:** 2026-04-28

**Authors:** Yuping Wan, Chen Xie, Qiang Wang, Xueping Wang

**Affiliations:** 1 Department of Neurology, The First Hospital of Lanzhou University, Lanzhou, China; 2 Pingbao Town Health Center, Baiyin, China

**Keywords:** autophagy, bilirubin, glutamate toxicity, inflammation, mitochondrial dysfunction, oxidation, Parkinson’s disease

## Abstract

Parkinson’s disease (PD) is the second most prevalent neurodegenerative disorder, significantly impacting patients’ quality of life. Although extensive research has focused on identifying biomarkers, PD diagnosis still relies heavily on clinical features. Current treatments are primarily symptomatic and fail to halt disease progression. Emerging evidence suggests that abnormal bilirubin (BR) levels correlate with PD severity and motor outcomes, highlighting BR’s potential as both a biomarker and a therapeutic target. This review elucidates the dual role of BR in PD pathogenesis—modulating oxidative stress, neuroinflammation, and mitochondrial dysfunction—and discusses novel BR-based therapeutic strategies.

## 1 Introduction

Parkinson’s disease (PD) is a prevalent and intricate neurological disorder, ranking as the second most common neurodegenerative condition after Alzheimer’s disease (AD), affecting over 1% of individuals aged 65 and older (Belin and Westerlund, 2008). Epidemiological data highlight the global ramifications of PD, with projections indicating that the number of people living with this condition will increase from 6.9 million in 2015 to an estimated 14.2 million by 2040 (Dorsey and Bloem, 2018). Age remains the most significant risk factor for developing PD, and men are more susceptible than women with a prevalence ratio of approximately 3:2 (Tolosa et al., 2021), thereby exacerbating personal, social, and economic burdens.

The neuropathological characteristics of PD include: (1) early and significant necrosis of dopaminergic neurons in the dense part of the substantia nigra pars compacta (SNpc), which can result in a mortality rate as high as 70%. As PD progresses, there is also a depletion of dopamine (DA) within the striatum (Zeng et al., 2018). (2) Formation of Lewy bodies: *α*-synuclein is insoluble and aggregates in the misfolded state to form intracytoplasmic inclusions. This abnormal accumulation and the subsequent spread of pathology between the gut, brainstem, and higher brain regions likely contribute to both the onset and progression of Parkinson’s disease (Morris et al., 2024).

Several potential mechanisms underlying the pathogenesis of PD have been proposed: (1) Oxidative Stress (OS): Under normal circumstances, a steady-state balance exists between oxidative and antioxidant systems within the body. However, when reactive oxygen species (ROS) are produced at a rate that surpasses the capacity of antioxidant defenses, oxidative stress occurs, leading to significant damage to DNA, proteins, and lipids. This oxidative stress not only directly contributes to cellular damage but also affects the activation of signaling pathways that result in cell death (Jayanti et al., 2021). (2) Mitochondrial dysfunction: Mitochondria play a crucial role in the neurodegenerative processes associated with PD through their involvement in cellular energy production and signaling pathways. Mitochondrial dysfunction, an early event in PD pathogenesis, is closely linked to the overproduction of ROS, which promotes the formation of soluble *α*-synuclein oligomers and insoluble fibrils (Morris et al., 2024; Jayanti et al., 2021). Additionally, genetic evidence suggests that an increase in mitochondrial DNA (mtDNA) mutations, deletions, or rearrangements may further exacerbate mitochondrial dysfunction and dopaminergic neuronal death among PD patients—thereby promoting neurodegeneration and elevating the risk of developing PD (Simon et al., 2004; Gu et al., 2002). (3) Immune and inflammatory mechanisms: Extensive clinical and experimental evidences support the involvement of immune and inflammatory mechanisms in the onset and progression of PD. Increased levels of pro-inflammatory cytokines and other proteins related to microglial activation have been observed in serum and cerebrospinal fluid (CSF) from PD patients compared to healthy controls, reinforcing systemic inflammation as a central concept in PD neurodegeneration (Zimmermann and Brockmann, 2022). Pathological protein α-synuclein activates Toll-like receptors on microglia, resulting in an increased production of pro-inflammatory cytokines and chemokines. This cascade ultimately leads to chronic neuroinflammation, which exacerbates neuronal dysfunction and loss (Kouli et al., 2019). (4) Protein homeostasis and lysosomal dysfunction: Defects in protein homeostasis and lysosomal dysfunction are widely recognized as contributing factors to various neurodegenerative diseases, including PD. The protein degradation system is an integral component of the cellular quality control machinery that eliminates non-essential misfolded or damaged proteins (Lehtonen et al., 2019). Dysfunction of the proteasome exacerbates protein aggregation in PD; under physiological conditions, misfolded α-synuclein can be cleared by both the ubiquitin-proteasome pathway (UPP) and the autophagy-lysosomal pathway (ALP). A diminished clearance capacity of these pathways has been identified as a major pathophysiological mechanism underlying neurodegeneration in PD (Picca et al., 2021). (5) Genetics: PD is recognized as a genetic disorder. Over the past three decades, it has been established that genetics contributes approximately 25% to the risk of developing PD (Day and Mullin, 2021). Genetic alterations influence molecular pathways such as *α*-synuclein proteostasis and degradation, mitochondrial function, oxidative stress, and neuroinflammation (Jayanti et al., 2021). Currently, studies have shown that SNCA, LRRK2, VPS35, EIF4G1, DNAJC13, and CHCHD2 are associated with autosomal dominant inheritance patterns for PD; conversely, Parkin, PINK1, and DJ-1 mediate autosomal recessive inheritance. Additionally, other genes related to PD—such as GBA1, ATP13A2, C9ORF72, FBXO7, PLA2G6, POLG1, SCA2, and SCA34—contribute variably to the progression of this disorder (Zeng et al., 2018; Kalia and Lang, 2015).

Bilirubin (BR) is an endogenous compound that is the ultimate breakdown product of heme metabolism and serves as a diagnostic marker for liver and blood disorders (Fevery, 2008). Traditionally regarded as metabolic waste, it is known to have toxic effects (Rao et al., 2006). However, in recent decades, accumulating evidences have highlighted that BR possesses antioxidant, anti-inflammatory, immunomodulatory, cytoprotective, and neuroprotective properties. Furthermore, it plays a role in signaling pathways associated with various diseases, including neurodegenerative disorders (Tatikolov et al., 2025). The relationship between bilirubin levels and PD remains a topic of debate and we reviewed researches on PD and bilirubin (Table 1). A study conducted by Jin et al. (2021) indicated that PD patients exhibited elevated serum BR levels. Similarly, Zhou et al. (2025) and Moccia et al. (2015) reported comparable findings, suggesting that higher BR levels may serve as markers for oxidative stress and the severity of the disease. Conversely, research by Qin et al. (2015) demonstrated reduced serum BR levels in PD patients. Additionally, a cross-sectional study revealed a significant inverse correlation between bilirubin levels and the prevalence of PD (Su et al., 2024). Songsomboon et al. (2020) investigation found a negative correlation between BR levels and the severity of symptoms in PD patients, while Macías-García et al. (2019) work highlighted an inverse relationship between bilirubin levels and disease progression among this population. In a case–control study focusing on Hoehn-Yahr stage I (HY I) patients, researchers identified a significant association between BR levels and motor asymmetry, indicating that BR primarily exerts its protective effects within this group (Kazemi et al., 2022). Furthermore, serum BR levels were positively correlated with UPDRS III scores, correlating with improved motor outcomes (Albillos et al., 2021).

**Table 1:** Summary of clinical trial methods, conclusions, and changes in bilirubin levels regarding PD.

Reference	Bilirubin	Method	Conclusion
Songsomboon et al. (2020)	TB↑ IDB↑	This study recruited 61 PD patients from the neurology outpatient clinic and a control group of 135 elderly individuals from a club. PD staging, as well as motor and non-motor functions, were determined using the Hoehn-Yahr (H&Y) scale and the Unified Parkinson’s Disease Rating Scale (UPDRS), respectively.	The high serum total bilirubin (TB) and indirect bilirubin (IDB) levels in PD patients when compared with controls, and the negative association between TB and IDB levels and PD severity, suggest the possible role of serum bilirubin in the pathogenesis of PD.
Li et al. (2019)	IBIL↓	The differences in bilirubin concentrations between 78 PD subjects and 78 controls were assessed, and the differences in IBIL concentrations between different motor subtypes (tremor-dominant (TD), intermediate (I), and postural instability with gait difficulty (PIGD)) as well as between motor subtypes and the control group were also evaluated.	PD patients had lower indirect bilirubin (IBIL) concentrations compared to controls. There was no significant difference in IBIL concentrations between PD males and PD females. IBIL concentrations had negative relationships with levodopa-equivalent daily dose (LEDD) and positive relationships with tremor score. IBIL concentrations were significantly lower for PIGD than for TD subtype. The lower IBIL concentrations in PD compared to controls were mainly driven by the PIGD patients.
Macías-García et al. (2019)	Bilirubin↑	Included 420 PD patients and 435 healthy controls. Bilirubin levels in both groups were compared using linear regression and multivariate analysis adjusted according to age and sex. Secondly, a case study with the PD cohort was carried out and bilirubin levels were correlated with current treatment, duration and severity of disease.	The bilirubin levels in patients with Parkinson’s disease are significantly higher than those in the control group, and it was found that bilirubin levels are negatively correlated with the course of the disease, with higher bilirubin levels in the early stages of the disease, decreasing as the condition progresses. Higher bilirubin concentrations were identified in PD patients with Hoehn & Yahr stage ≤3.
Lee et al. (2019)	Bilirubin↑	Included 15 drug-naive early idiopathic Parkinson disease (IPD) patients and 62 essential tremor (ET) patients. PET images were analyzed using volume-of-interest templates for 12 striatal subregions and 1 occipital area, and the specific-to-nonspecific binding ratio (SNBR) was calculated.	Levels of bilirubin were significantly higher in the IPD group than in controls, and bilirubin level was the factor showing the most correlations with SNBR in IPD. Furthermore, levels of bilirubin showed a positive correlation with SNBR in more affected posterior putamen in the IPD group, but a negative one in the ET group.
Qin et al. (2015)	IBIL↓	This study included 425 PD patients and 460 controls, aiming to explore the correlation between bilirubin and uric acid (UA) levels and Parkinson’s disease (PD) symptoms in the Chinese population.	Compared to controls, indirect bilirubin (IBIL) and UA concentrations were lower in PD patients. Serum IBIL in different age subgroups and H&Y stage subgroups were also lower compared to the control group but were not significantly different among these subgroups. Females in the control group had significantly lower serum IBIL and UA concentrations than males. In early PD (patients with 2-year medical history and no treatment), serum IBIL and UA concentrations were also lower than the controls.
Moccia et al. (2015)	Bilirubin↑	A cross-sectional case–control study was conducted to evaluate the differences in bilirubin levels between 75 newly diagnosed, drug-naive Parkinson’s disease patients and 75 control subjects. Subsequently, the PD patients were enrolled in a two-year longitudinal study to assess the relationship between disease progression and baseline bilirubin levels.	Parkinson’s disease subjects showed higher levels of bilirubin compared with controls. Slightly worse motor symptoms were found in PD patients with higher bilirubin levels. At 2-year follow-up evaluation, PD subjects with higher bilirubin levels required fewer dopaminergic drugs.
Scigliano et al. (1997)	Bilirubin↑	By investigating 255 patients with idiopathic Parkinson’s disease from 1976 to 1989, among whom 162 received levodopa treatment, 93 started using levodopa during hospitalization, and a control group consisting of 224 other patients, the bilirubin levels and other blood variables were compared between the Parkinson’s disease group and the control group.	The study found a highly significant (about 20%) increase in plasma bilirubin in 162 PD patients on chronic L-dopa treatment compared to 93 untreated parkinsonians and 224 non-parkinsonian controls.
Zhou et al. (2025)	Total bilirubin↑	By analyzing 1,631 participants from 2010 to December 11, 2024, the association between total bilirubin and early Parkinson’s disease was assessed.	Total bilirubin levels were significantly and independently linked to a 16% heightened risk of early-stage PD. Bilirubin levels were found to be negatively correlated with DAT-scan values in the putamen and striatum regions.
Su et al. (2024)	STB↓	A cross-sectional analysis was conducted using data from 25,637 participants in the National Health and Nutrition Examination Survey (NHANES) from 1999 to 2018, aiming to clarify the relationship between serum total bilirubin (STB) levels and Parkinson’s disease in the U. S. population.	The level of STB is significantly negatively correlated with the prevalence of PD.
Kazemi et al. (2022)	TB↑	This study aimed to investigate the association of total bilirubin (TB) with motor signs and asymmetry in different stages of early PD. A case–control study was performed to investigate the differences in TB levels in PD patients and healthy controls (HC) both carrying LRRK2 variants.	TB concentrations were found to have significantly increased in the PD population compared to HC. A significant relationship was found between TB and motor asymmetry in HY I patients.
Albillos et al. (2021)	Bilirubin↑	By analyzing 35 age-matched controls, 29 NOVO-PD, 35 PD and 38 essential tremor (ET) patients, the study examined the plasma metabolomics profiling and their association with motor and non-motor symptoms (NMS) in patients with PD, and to determine differences between *de novo* PD compared to moderate-advanced PD vs. controls and patients with ET.	Bilirubin presented good predictive accuracy for differentiating de novo PD and advanced PD from controls and ET. Higher plasma or serum bilirubin and/or biliverdin contents have been observed in PD patients, with positive correlations of serum bilirubin levels with UPDRS III, along with better motor outcomes.

Regarding the “bidirectional effect” of bilirubin in PD, it suggests that the biological effects of bilirubin are not singular, but show obvious concentration dependence and time dependence. Based on this, study hypothesize that acute, mild elevations in bilirubin may be a compensatory protective response of the body in the face of neuroinflammation and oxidative stress, helping to delay neuronal damage (Macías-García et al., 2019; Lee et al., 2019). In the early stages of PD, the body promotes bilirubin production through heme oxygenase-1 (HO-1) overexpression to counteract oxidative stress damage. When bilirubin metabolism is chronically imbalanced or becomes extremely elevated, it can exceed the body’s regulatory capacity and turn toxic, worsening dopaminergic neuron damage and disease progression. In the middle to late stages of PD, continuous HO-1 overexpression of bilirubin exacerbates neurodegeneration, while in the terminal stage, due to the depletion of the body’s antioxidant system, bilirubin levels actually decrease. This kind of “protection-damage” dynamic transformation can well explain the phenomenon of the “bilirubin paradox” in PD (Moccia et al., 2015; Albillos et al., 2021).

The heterogeneity of bilirubin levels in PD patients across different studies is mainly shaped by multiple multidimensional confounding factors. The difference in the distribution of disease stages is the primary reason. Early-stage PD often manifests as a compensatory increase in bilirubin, whereas in the middle and late stages, bilirubin levels may decrease due to the depletion of the body’s antioxidant system. The proportion of early, middle and late-stage patients varies across different studies, which directly leads to contradictory research results (Zhou et al., 2025; Su et al., 2024). Secondly, medication status may affect bilirubin levels. Scigliano et al. (1997) reported that PD patients who received long-term levodopa treatment had elevated plasma bilirubin levels, showing a protective effect, whereas in most studies, treatment of PD patients was not recorded, making it impossible to adjust for drug effects, thus leading to bias in cross-sectional comparisons (Songsomboon et al., 2020). Third, genetic background is crucial. Genetic variations in the UGT1A1 and HMOX1 genes directly affect the synthesis, metabolism, and clearance of bilirubin, which may lead to differences in baseline bilirubin levels among individuals as well as susceptibility to PD (Macías-García et al., 2019). Fourth, demographic factors including age, gender, BMI, smoking, drinking, and ethnicity can affect bilirubin metabolism and may explain the results of studies in specific populations. For example, a study by Qin et al. (2015) in a Chinese cohort showed a decrease in bilirubin levels, whereas European studies showed an increase in bilirubin levels (Su et al., 2024; Kazemi et al., 2022). Fifth, interference from comorbidities also contributed to the critical results. PD patients are usually older and often have one or more comorbidities. Reduced serum bilirubin concentration is associated with various diseases, including metabolic syndrome, diabetes, cardiovascular diseases, and cancer (Qin et al., 2015; Li et al., 2019). Finally, differences in the selection of detection indicators (total bilirubin, direct bilirubin, or indirect bilirubin), sample size, study design (cross-sectional or longitudinal), and the relationship between sample collection time and the onset of the disease further increase the complexity of cross-study comparisons. These reasons may explain the contradictions and inconsistencies in bilirubin levels observed in clinical studies of PD patients.

While previous reviews have examined bilirubin’s antioxidant properties in isolation or discussed oxidative stress in PD broadly, the present review offers several unique contributions. Firstly, we provide an integrated mechanistic framework linking bilirubin to five interconnected pathogenic pathways in PD, such as oxidative stress, inflammation, mitochondrial dysfunction, glutamate excitotoxicity, and autophagy. Secondly, we critically evaluate the dual role of bilirubin, distinguishing between its protective effects at physiological concentrations and its potential toxicity at elevated levels, a nuance often underemphasized in prior literature. Thirdly, we synthesize emerging evidence on bilirubin-based therapeutic strategies, including novel pharmacological modulators (e.g., BRUP-1), nanotechnology-based delivery systems, and UGT1A1-targeted approaches. Finally, we propose a conceptual model illustrating how bilirubin serves as a central homeostatic mediator integrating multiple neuroprotective pathways. This integrative perspective aims to advance beyond descriptive accounts toward a mechanistic understanding that may inform future therapeutic development.

## 2 The heme catabolic pathway

**Figure 1:**
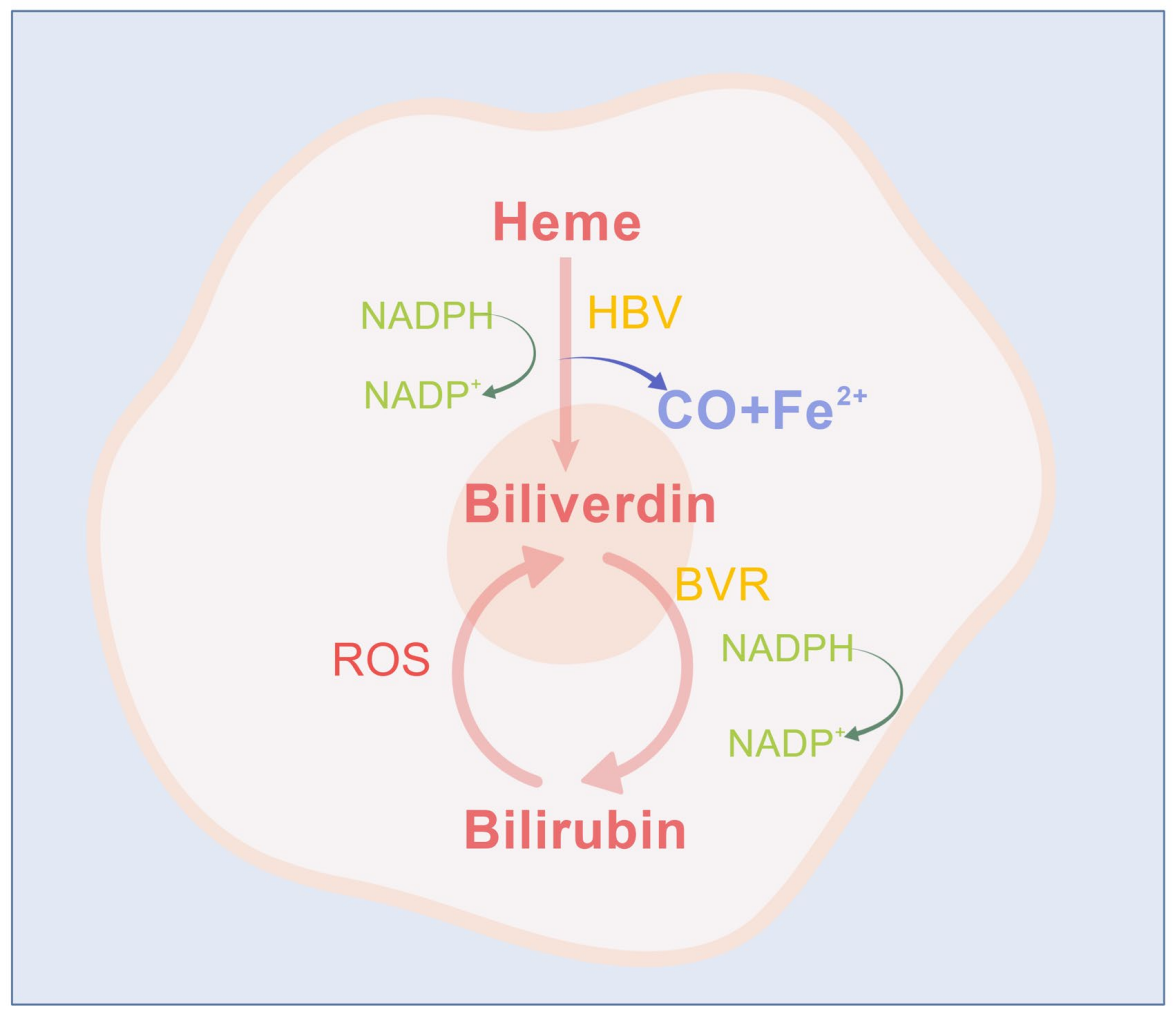
The metabolic diagram of bilirubin in the body.

Heme is a cyclic tetrapyrrolic compound characterized by a centrally bound iron atom. The primary source of free heme is hemoglobin derived from senescent red blood cells, and heme metabolism takes place in all mammalian cells (Figure 1). In the presence of oxygen and the reducing equivalents provided by NADPH-cytochrome P-450 oxidoreductase, heme oxygenases (HO-1 and HO-2) catalyze the oxidation and cleavage of heme at the *α*-meso-carbon bridge, yielding equimolar amounts of ferrous iron (Fe^2+^), carbon monoxide (CO), and biliverdin-IXα (BV). BV undergoes further reduction at C10 (*γ* bridge) by biliverdin reductase (BVR), which requires NAD(P)H, resulting in bilirubin-IXα (BR) as the final product. Conversely, BR can generate BV through reactive oxygen species (ROS) (Mancuso, 2025; Pentón-Rol et al., 2021; Vítek and Schwertner, 2007). Among these, HO, BV, BVR, and BR are classified as yellow players (YPs). YPs serve as a homeostatic and defensive mechanism within cells that can act directly or indirectly—through signaling pathways—on key organisms, thereby enhancing the self-protective potential of neuronal cells under stressful conditions (Jayanti et al., 2021; Jayanti et al., 2023). It is widely acknowledged that free heme can be toxic due to its pro-oxidant effects; thus, it is preferable to eliminate it from the body promptly. However, there is now a broad consensus that the induction of heme catabolism represents an adaptive and ultimately protective response to oxidative injury (Salinas et al., 2003).

The BR is a final product of heme metabolism that exhibits significant antioxidant properties, likely making it the most abundant endogenous antioxidant found in mammalian tissues. It effectively scavenges not only ROS but also reactive nitrogen species (RNS), reduces superoxide production, and inhibits glutamate excitotoxicity. Notably, BR demonstrates neuroprotective effects even at nanomolar concentrations and can counteract intracellular concentrations of H_2_O_2_ that are 10,000 times higher—making it more potent than α-tocopherol (Mancuso et al., 2012; Jayanti et al., 2020; Jayanti et al., 2022; Doré et al., 1999; Mancuso, 2021). Calculations suggest that BR can traverse the blood–brain barrier, thereby exerting substantial antioxidant effects within the brain (Scigliano et al., 1997). Beyond its antioxidant activity, BR possesses other critical biological properties including anti-inflammatory, immunomodulatory, cytoprotective, and neuroprotective effects (Tatikolov et al., 2025). BR has been demonstrated to exhibit greater anti-inflammatory activity compared to dexamethasone (Jayanti et al., 2022). In related models of islet isolation and hypoxic stress, the supplementation of exogenous BR significantly reduced the release of damage-associated molecular patterns (DAMPs), such as HMGB1, along with inflammatory cytokines like IL-1β and IL-6, as well as chemokines including MCP-1. These findings highlight the cytoprotective and anti-inflammatory properties of BR (Adin et al., 2017). Recent studies have also revealed that BR possesses potent immunomodulatory effects by modulating both innate and adaptive immune responses. While high concentrations of BR can be cytotoxic, moderate increases in its levels are associated with immunosuppressive effects (Jangi et al., 2013). Furthermore, BR activates various nuclear and cytoplasmic receptors, mimicking the endocrine functions typical of hormonal substances (Vítek, 2020). Some research suggests that BR may exert a hormonal effect through direct binding to and activation of the nuclear receptor peroxisome proliferator-activated receptor-alpha (PPARα), which subsequently acts as a signaling molecule driving gene transcriptional changes in physiological responses (Stec et al., 2022). Additionally, although high concentrations (>250–300 μM in serum) may confer neuroprotective benefits, they could also disrupt lipid membranes within the brain and accumulate in specific regions, potentially leading to neurotoxicity associated with kernicterus due to the highly hydrophobic nature of bilirubin (Tatikolov et al., 2025; Doré et al., 1999; Cuadrado and Rojo, 2008).

## 3 The BR in Parkinson’s disease (PD)

**Figure 2:**
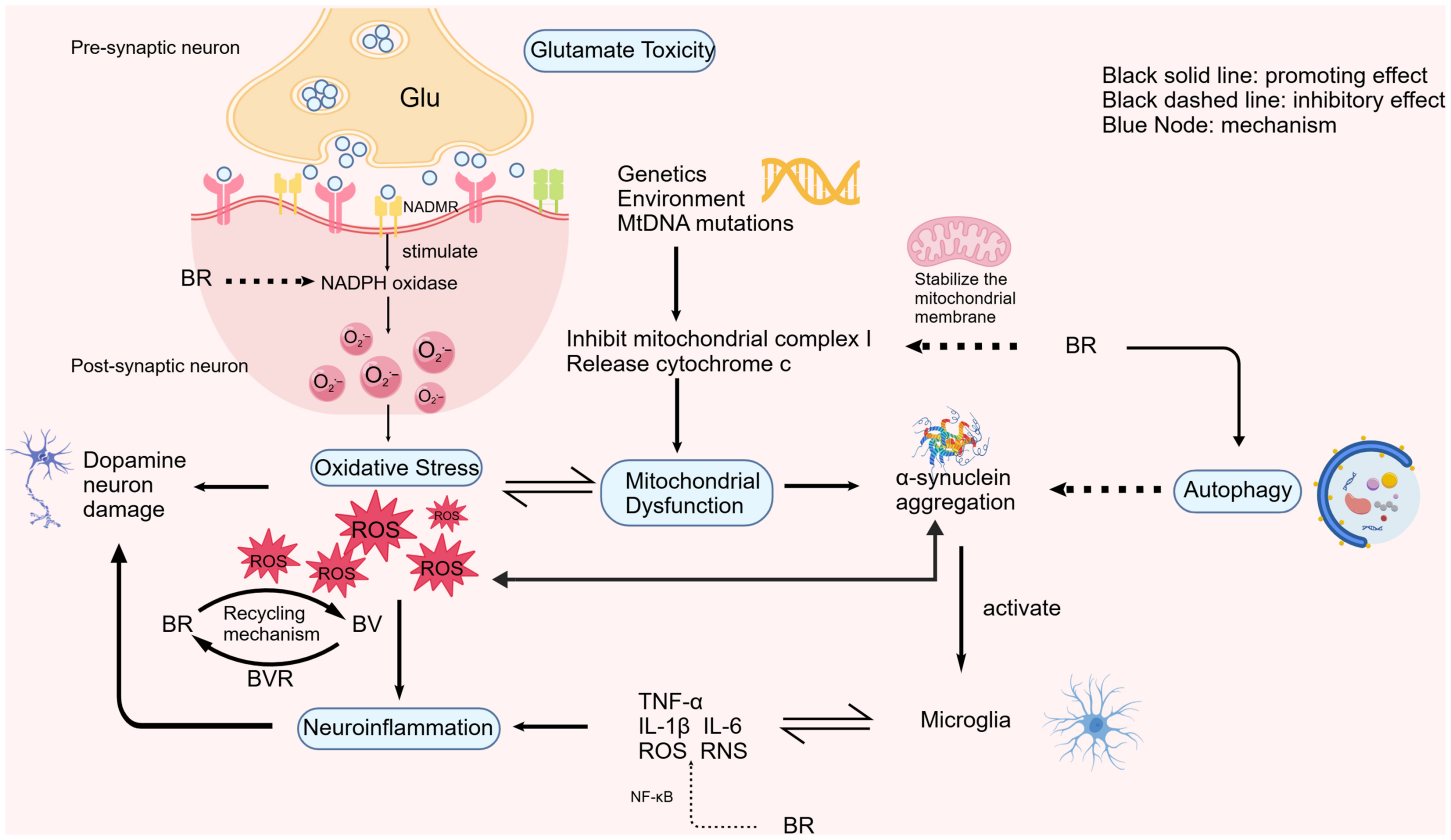
Bilirubin is simultaneously involved in multiple pathological processes in Parkinson's disease. Oxidative stress lies at the heart of this network: mitochondrial dysfunction leads to the production of excessive ROS, which not only directly damage dopaminergic neurons but also trigger neuroinflammatory responses and promote the aggregation of α-synuclein. Bilirubin interrupts this cascade reaction at several points: (1) By directly scavenging ROS via a recycling mechanism, thereby reducing oxidative damage and breaking the positive feedback loop in which oxidative stress exacerbates mitochondrial dysfunction; (2) By inhibiting NF-κB signaling and downregulating TNF-α, IL-1β, and IL-6, bilirubin can alleviate neuroinflammation, thereby reducing microglia-mediated neuronal damage; (3) By stabilizing the mitochondrial membrane and reducing the release of cytochrome c, bilirubin inhibits mitochondrial dysfunction; (4) By inhibiting NADPH oxidase, bilirubin reduces glutamate-induced superoxide production, thereby alleviating excitotoxicity; (5) Furthermore, bilirubin activates autophagy, promoting the clearance of aggregated α-synuclein, thereby breaking this bidirectional pathogenic cycle, namely, the cycle in which protein aggregation exacerbates oxidative stress and neuroinflammation.

Bilirubin may be involved in the pathogenesis of PD through oxidative stress, inflammation, mitochondrial dysfunction, glutamate toxicity, and autophagy. Moreover, these pathological mechanisms are not independent and can affect each other, thus we summarized these pathological mechanisms in a conceptual figure (Figure 2). In addition, how bilirubin affects the pathogenesis of Parkinson’s disease through different mechanisms is described in detail.

### 3.1 Oxidation

**Figure 3:**
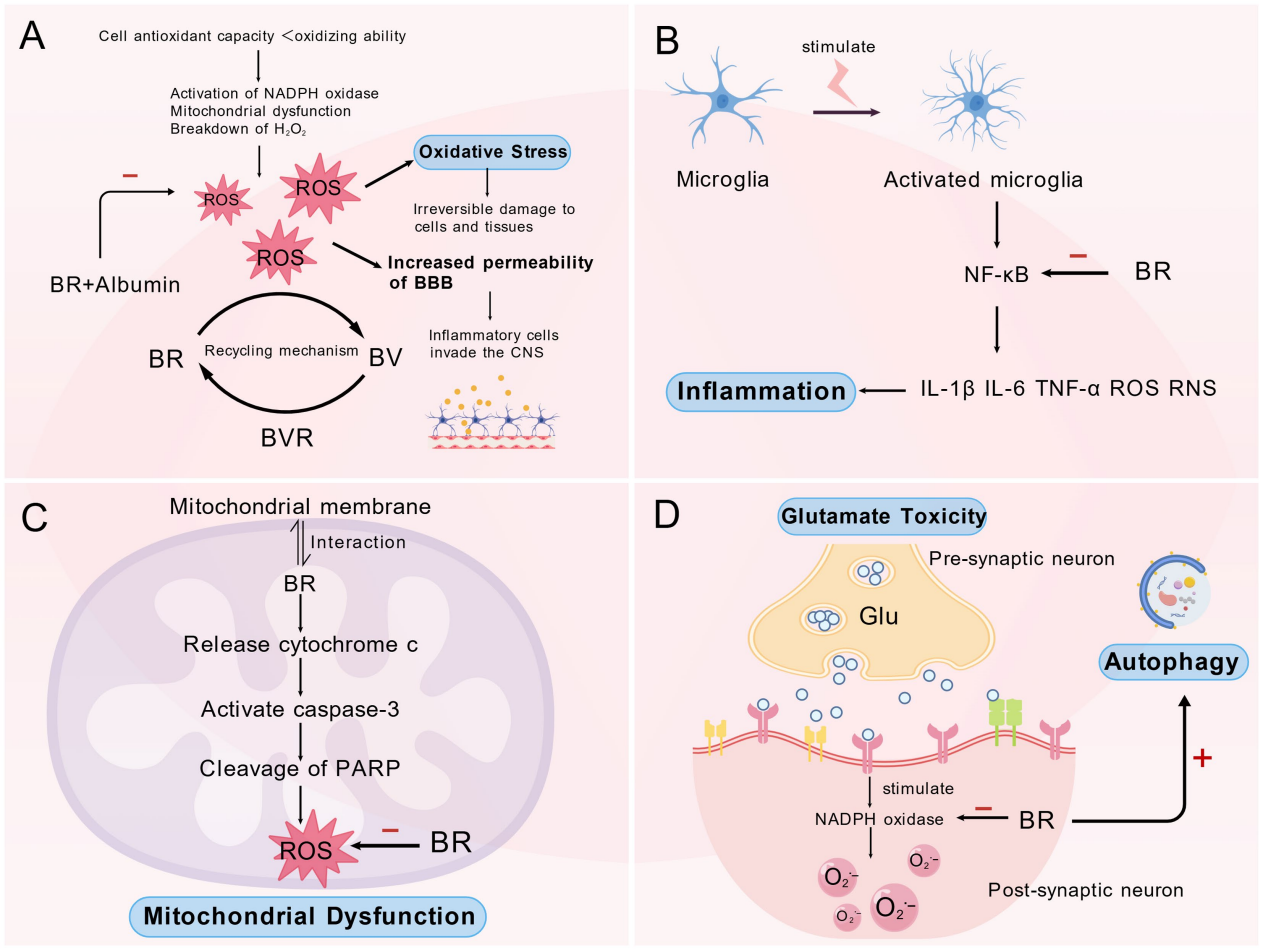
The potential mechanisms underlying bilirubin's involvement in the pathogenesis of Parkinson's disease. **(A)** Oxidative stress constitutes a plausible pathway by which BR may exert neurotoxic effects in Parkinson's disease. **(B)** BR may contribute to the pathogenesis of Parkinson's disease via neuroinflammatory process. **(C)** Mitochondrial dysfunction may serve as a mechanistic link between BR and the progression of Parkinson's disease. **(D)** Glutamate excitotoxicity and dysregulation of autophagy as potential underlying mechanisms through which BR may affect the development of Parkinson's disease.

The ROS is regarded as a crucial modulator in the progression of PD, and its accumulation constitutes a significant aspect of numerous detrimental molecular pathways observed during the early stages of PD, prior to the onset of neuronal death (Trist et al., 2019). To mitigate damage induced by free radicals, YPs play an essential role due to their well-documented capacity to eliminate both ROS and RNS. Notably, the antioxidant properties of BR are particularly vital in the nervous system as it serves as a key molecule at the intersection of protecting the body against ROS (Figure 3A) (Breimer and Mikhailidis, 2010).

The BR is a natural antioxidant with notable properties that combat oxidative stress. It interacts with ROS to produce hydrophilic oxidative metabolites, which are ultimately excreted in urine. This process has been shown to mitigate cellular damage induced by ROS in numerous *in vitro* and *in vivo* studies (Shibama et al., 2019; Mancuso et al., 2006; Hooda et al., 2017). It is considered that BR is an effective scavenger of peroxide radicals, with in vitro micromolar concentrations demonstrating its ability to efficiently neutralize peroxyl radicals generated chemically, whether in homogeneous solutions or multilamellar liposomes (Stocker et al., 1987; Bulmer et al., 2008). This efficacy stems from the antioxidant properties of BR, which involve its oxidation to BV. The presence of a substantial amount of excess BVR facilitates the rapid regeneration of BR. This cyclic regeneration mechanism can amplify the antioxidant effect of BR by 10,000 times or more, enabling low micromolar concentrations to effectively eliminate millimolar levels of toxic oxidants. Research indicates that bilirubin at concentrations as low as 10 nM can protect against H_2_O_2_ at concentrations up to 10,000 times higher (Vitek and Ostrow, 2009; Sedlak et al., 2009). Unconjugated bilirubin effectively scavenges singlet oxygen, interacts with superoxide anions and peroxyl radicals, and acts as a reducing substrate for peroxidases in the presence of hydrogen peroxide or organic hydroperoxides (Stocker and Ames, 1987). Under physiological conditions, the majority of bilirubin binds to albumin, enhancing its antioxidant properties; specifically, one mole of bilirubin can bind to plasma albumin and eliminate two moles of oxygen free radicals. This mechanism helps prevent tissue damage and lipid peroxidation resulting from excessive ROS production (Chen et al., 2020; Sameiro-Faria et al., 2014). Furthermore, physiologically relevant concentrations of bilirubin provide protection against protein- and lipid-induced myeloperoxidase-generated hypochlorous acid oxidation, suggesting that this inhibition is accompanied by direct scavenging of chloramines by albumin-bound bilirubin (Boon et al., 2015). Epidemiological studies have notably indicated that lipids serve as markers for the severity of PD, with low-density lipoprotein (LDL) concentrations potentially increasing the incidence of PD. Under physiological conditions, BR, recognized as an effective lipid chain-breaking antioxidant, can inhibit LDL lipid peroxidation and prevent the formation of oxidized LDL. This action protects low-density lipoprotein cholesterol (LDL-c) and other lipids from oxidative damage caused by reactive oxygen species, thereby reducing both the risk and incidence of PD (Chen et al., 2020; Sameiro-Faria et al., 2014; Fu et al., 2020; Xu et al., 2024).

Lee et al. (2019) conducted a study involving 105 patients with drug-naive, early-stage idiopathic Parkinson disease (IPD) and 62 patients with essential tremor (ET), demonstrating that bilirubin was the antioxidant marker most significantly associated with dopaminergic deficit, as measured by [18F] FP-CIT PET/CT, in patients with early-stage drug-naive IPD. The findings suggest that elevated bilirubin levels may represent a compensatory response to counteract oxidative stress during the early stages of IPD progression (Lee et al., 2019). Takahashi et al. (2000) demonstrated that cortical neurons cultured from mice harboring the Swedish mutation associated with Alzheimer’s disease exhibited impaired BR production, leading to increased hydrogen peroxide toxicity. Fuhua et al. (2012) performed a study involving 388 participants, comprising 97 individuals diagnosed with myasthenia gravis (MG), 135 with multiple sclerosis (MS), and 156 healthy controls. The researchers observed that serum levels of total, direct, and indirect bilirubin were significantly lower in MG patients compared to the healthy control group (*p* = 0.001, *p* = 0.012, and *p* = 0.001, respectively), suggesting an association between MG and the antioxidant properties of bilirubin (Fuhua et al., 2012). In a study of patients with AD, the levels of bilirubin and its derivatives in cerebrospinal fluid were significantly elevated compared to those in the control group (*p* = 0.0008). These findings suggest that the accumulation of bilirubin metabolites in the brains of individuals with AD may reflect a compensatory response to chronic oxidative stress (Kimpara et al., 2000). In an *ex vivo* experiment using isolated aortic rings obtained from untreated ApoE-deficient mice, bilirubin was found to functionally mimic heme in the vascular tissue *in vitro*. It effectively inhibited NADPH oxidase activity within the concentration range of 0.01–1 μmol/L, interfered with agonist-induced assembly of the NADPH oxidase enzyme complex in the cell membrane, and consequently attenuated enzyme activation, leading to a reduction in oxidative stress (Datla et al., 2007).

Collectively, these studies indicate that bilirubin can effectively reduce ROS levels, demonstrate substantial antioxidant properties, maintain cellular function, and alleviate oxidative stress in PD and other neurological disease and have the potential to a treatment target of PD.

### 3.2 Inflammation

In recent years, inflammation has emerged as a significant causative factor in the pathogenesis and progression of PD, often preceding observable neuronal damage and degeneration (Zimmermann and Brockmann, 2022; Isik et al., 2023). A longitudinal study demonstrates that in patients with PD, a pro-inflammatory profile—characterized by elevated levels of TNF-α, IL-1β, IL-2, and IL-10—is associated with lower Mini-Mental State Examination (MMSE) scores and more rapid motor decline. In contrast, an anti-inflammatory profile is correlated with better cognitive performance and greater stability in motor function (Figure 3B) (Williams-Gray et al., 2016).

A growing body of evidence indicates that BR exhibits substantial anti-inflammatory activity by suppressing the inflammatory response through inhibition of pro-inflammatory cytokines, including TNF-α, IL-1β, and IL-6, thus contributing to the preservation of neuronal integrity (Jayanti et al., 2023; Li et al., 2020). Research suggests that BR can attenuate inflammatory responses by inhibiting NF-κB-mediated cytokine production and reducing the generation of ROS and RNS (Lee and Choi, 2018; Fernandes et al., 2006). Moreover, BR induces ER stress in neuronal cells through the PERK and IRE1 pathways, playing a significant role in neuronal inflammatory responses and apoptosis (Qaisiya et al., 2017). Studies indicate that BR acts as an aryl hydrocarbon receptor (AhR) ligand; AhR serves as an anti-inflammatory factor in macrophages that regulates AhR-dependent gene expression pathways (Phelan et al., 1998; Nguyen et al., 2013). On the other hand, BR protects the blood–brain barrier (BBB) from alterations in permeability induced by free radicals, thereby preventing the infiltration of inflammatory cells into the central nervous system (CNS) (Liu et al., 2003; Peng et al., 2011). Jayanti et al. (2022) utilized organotypic brain cultures of the substantia nigra (OBCs-SN) to establish an ex vivo progressive PD model and examined the neuroprotective effects of low-concentration unconjugated bilirubin (UCB, ranging from 0.5 to 4 μM). Their findings demonstrated that UCB at concentrations of 0.5 and 1 μM completely prevented the loss of tyrosine hydroxylase-positive (TH^+^) dopaminergic neurons (DOPAns) by modulating the expression of the key inflammatory mediator TNF-α, thereby elucidating the anti-inflammatory properties of UCB (Jayanti et al., 2022). Mao et al. (2025) employed an inflammatory model to show that bilirubin binding to with-no-lysine kinase 1 (WNK1) effectively inhibited lipopolysaccharide (LPS)-induced activation of the NLRP3 inflammasome, resulting in anti-inflammatory effects in murine neurons. A study utilizing a spinal cord injury (SCI) model demonstrated that bilirubin treatment facilitates functional recovery through the upregulation of Gas6-Axl signaling, leading to increased SOCS3 expression and downregulation of pro-inflammatory mediators, including IL-1β and MMP-9. These molecular changes were associated with improved motor function in SCI-induced mice. Furthermore, bilirubin treatment attenuated microglial activation, underscoring its neuroprotective and anti-inflammatory effects (Jiang et al., 2025).

The above evidence suggests that BR is beneficial for inflammation and plays an important role in the inflammatory process, and BR appears to be an important auxiliary factor in the progression of PD.

### 3.3 Mitochondrial dysfunction

Mitochondrial dysfunction has been demonstrated to be a key pathogenic mechanism in multiple neurodegenerative diseases, including PD (Figure 3C) (Beal, 2005).

At slightly elevated unbound concentrations, BR exhibits toxicity toward astrocytes and neurons, leading to damage of mitochondria and plasma membranes. Consequently, mitochondria have long been regarded as particularly vulnerable to BR-mediated toxicity (Ostrow et al., 2004). BR functions as a multi-site respiratory inhibitor within the mitochondria; on one hand, it acts as a decoupler for oxidative phosphorylation independent of the traditional decoupling mechanisms (Noir et al., 1972). On the other hand, animal studies suggest that BR interacts with the mitochondrial membrane, enhancing its permeability and altering lipid and protein properties, redox status, and cytochrome c content. The release of cytochrome c triggers caspase-3 activation and subsequent cleavage of poly (ADP) ribose polymerase (PARP), which inhibits electron transfer in the respiratory chain while further increasing in ROS production—ultimately inducing cell death. Thus, it appears that mitochondria mediate BR-induced apoptosis (Rodrigues et al., 2002a,b; Rodrigues et al., 2000). Some researchers have reported that at specific doses, BR can selectively stabilize the mitochondrial membrane during NLRP3 inflammasome activation; this positions it as a potential mitochondrial-targeted therapeutic agent against inflammasome-related diseases in future applications (Li et al., 2022).

### 3.4 Glutamate toxicity

Glutamate is regarded as the most important excitatory amino acid neurotransmitter in the brain (Willard and Koochekpour, 2013; Silva et al., 1999). Extensive clinical and experimental evidence supports the involvement of glutamate-induced excitotoxicity in the onset and progression of PD (Figure 3D) (Abdik and Cakir, 2021; Gautam et al., 2023; Ribeiro et al., 2017; Das et al., 2025).

The BR interferes with the uptake of glutamate into synaptic vesicles, astrocytes, and neurons, resulting in elevated extracellular glutamate levels and excessive activation of glutamate receptors. This can lead to cellular swelling and ultimately induce cell death through apoptosis and necrosis. The accumulation of extracellular glutamate is neurotoxic, while astrocytes play a protective role by removing excess glutamate from the extracellular space (Barateiro et al., 2014; Silva et al., 2012; Fernandes et al., 2004). Moreover, within the brain, O₂^._^ acts as a vital neuromodulator. Activated glutamatergic *N*-methyl-d-aspartic acid (NMDA) receptors (NMDARs) stimulate NADPH oxidase to produce O₂^._^, while bilirubin and related tetrapyrrole compounds mitigate O₂^._^ levels and signal transduction by inhibiting NADPH oxidase activity (Vasavda et al., 2019). Grojean et al. (2001) demonstrated through neuronal cell cultures that the neurotoxic effects of bilirubin require the involvement of glutamate toxicity. Low concentrations of unconjugated bilirubin may further enhance glutamate neurotoxicity by activating NMDA receptors, thereby promoting apoptosis and exacerbating hypoxic injury in the developing brain. Additionally, bilirubin may sensitize neurons to glutamate toxicity through its direct influence on protein kinase C (Grojean et al., 2001).

Therefore, we can conclude that bilirubin plays a significant role in the progression of PD by accelerating dopaminergic neuron death in the substantia nigra reticulata due to excitotoxicity induced by elevated extracellular glutamate and more studies are needed to verify this conclusion.

### 3.5 Autophagy

Autophagy is one of the two key cellular processes responsible for cellular clearance and the maintenance of homeostasis (Parzych and Klionsky, 2014; Kinet and Dehay, 2023). Impaired autophagy has been shown to contribute to the pathogenesis of PD by not only dysregulating the functions of proteins encoded by PD-associated genes but also hindering the clearance of α-synuclein. This results in its accumulation and subsequent misfolding—creating a bidirectional pathogenic loop (Figure 3D) (Hou et al., 2020; Erekat, 2022).

A study reveals that UCB induces autophagy in SH-SY5Y neurons and U87 astrocytic glial cells through the mTOR/ER-stress/PKC/calcium signaling pathway. Furthermore, preactivation of autophagy offers protection to the SH-SY5Y neuronal cell line, primary hippocampal neurons, and U87 astrocytoma cell line against UCB cytotoxicity (Qaisiya et al., 2017). In a rat model of non-alcoholic fatty liver disease (NAFLD), it was found that bilirubin promotes autophagic flux by upregulating the expression of various autophagy-related genes (Atgs), including Atg3, Atg5, Atg6/Beclin-1, and Atg7, thereby mitigating the risk of NAFLD or slowing its progression (Tavakoli et al., 2024). Palmela et al. (2012) demonstrated that prolonged exposure to UCB has significant effects on the transendothelial properties of human brain microvascular endothelial cells (HBMECs). This exposure alters both the structural integrity of intercellular junctions and protein expression levels, while also triggering cellular responses to stress stimuli, as evidenced by increased autophagy (Palmela et al., 2012). Moreover, gene expression data and experimental validation suggest that exposure to UCB leads to the upregulation of autophagy-related genes in human neuroblastoma SH-SY5Y cells (Calligaris et al., 2009). The studies mentioned above have established that bilirubin has the potential to activate autophagy. However, whether it contributes to increased autophagy during the progression of PD necessitates further experimental investigation.

## 4 Future prospective: bilirubin as a treatment in PD

Over the past few decades, significant efforts have been dedicated to researching and developing effective treatments for PD. However, PD remains an incurable condition. Current therapies primarily aim to enhance DA function in order to alleviate disease symptoms; nevertheless, no treatment has been identified that can halt or slow neuronal degeneration (Armstrong and Okun, 2020). Furthermore, pathological changes beyond the motor system can lead to cognitive, autonomic, and psychiatric symptoms and are inadequately addressed by existing therapies (Savitt et al., 2006). Consequently, there is an urgent need for innovative therapeutic strategies aimed at halting or slowing selective neuronal degeneration.

In recent years, a variety of innovative therapeutic strategies have been proposed: Cell therapy, such as mesenchymal stem cells (Riecke et al., 2015), photobiomodulation (Hamilton et al., 2019; Liebert et al., 2024), other medications, such as Exenatide (Church, 2021), acupuncture (Zhao et al., 2021), gene therapy (Buttery and Barker, 2020), and non-invasive brain stimulation, including transcranial magnetic stimulation and transcranial direct current stimulation (Lee et al., 2024; Giustiniani et al., 2025).

The potential to identify the risk of neurological, cardiac, or metabolic diseases through a simple blood sample is an intriguing prospect. Discussions regarding the therapeutic applications of BR have commenced, grounded in its biological properties such as antioxidant, anti-inflammatory, immunomodulatory, cytoprotective, and neuroprotective effects. Research indicates that mildly elevated levels of BR can offer protection against various diseases linked to increased oxidative stress, including cardiovascular disease (CVD), diabetes, cancer, and metabolic syndrome (Nano et al., 2016; Gazzin et al., 2016). In certain circumstances, elevated levels of beta-hydroxybutyrate may pose potential neurotoxicity to the central nervous systems of both term and preterm infants. Significantly high bilirubin levels can result in bilirubin encephalopathy, leading to kernicterus and severe, permanent neurodevelopmental impairments (Dani et al., 2019). However, extensive research suggests that mild elevations in bilirubin levels may be beneficial. Administering bilirubin within non-toxic ranges could provide a protective effect. For instance, one study demonstrated that bilirubin applied in a xenograft tumor model might contribute to cancer defense by interfering with pro-carcinogenic signaling pathways, effectively suppressing tumor growth (Ollinger et al., 2007). Furthermore, elevated BR concentrations may help inhibit plaque formation and the subsequent development of atherosclerosis. Serum BR exhibits a protective effect against CVD and related conditions, with UGT1A1 being the primary gene responsible for regulating serum bilirubin levels (Lin et al., 2010). Animal studies have indicated that serum BR concentrations can affect body fat distribution in diabetic individuals. By alleviating visceral obesity and reducing inflammation in adipose tissue, it helps prevent insulin resistance, thereby emerging as a promising therapeutic target for safeguarding those with visceral obesity and diabetes from insulin resistance (Takei et al., 2019).

Research suggests that PD patients with elevated BR levels require lower doses of dopaminergic medications and demonstrate reduced motor scores during the off phase (Moccia et al., 2015). Concurrently, plasma BR levels are found to be increased in PD patients receiving long-term levodopa therapy (Scigliano et al., 1997). These findings indicate that BR serves not only as a biomarker for the progression of PD but also as a potential therapeutic target.

Although bilirubin shows promising potential for the treatment of PD, a number of translational challenges still need to be addressed before it can be put into clinical use. Firstly, the narrow therapeutic window poses a significant challenge: while bilirubin exhibits potent antioxidant, anti-inflammatory, immunomodulatory, cytoprotective and neuroprotective effects at therapeutic concentrations (~20–200 μM), elevated levels (serum concentrations >250–300 μM) carry a risk of neurotoxicity and may damage brain regions such as the basal ganglia and brainstem; consequently, a precise dosing strategy is required (Tatikolov et al., 2025; Kaur et al., 2025). Secondly, bilirubin’s low aqueous solubility, the potential toxicity associated with hyperbilirubinemia, its relatively low bioavailability and susceptibility to oxidation limit its clinical application (Chen et al., 2020; Yao et al., 2020b). To date, the products used in research studies are chemical grade and have been universally animal-derived, carrying the potential for transmitting prions or viral infectious agents. Commercially available bilirubin is porcine in origin and is composed of mixed isomers, and in its current form, it is unsuitable for use in human subjects (Adin, 2021). Finally, UGT1A1, encoding the uridine diphosphate glucuronosyltransferase (UGT) 1A1 enzyme, participates in the elimination of BR (Nelson et al., 2021). UGT1A1 promoter and coding sequence gene variants decrease hepatic bilirubin conjugation and affect the process of bilirubin glucuronidation (Chávez-Peña et al., 2020). So far, 130 UGT1A1 mutations in both coding and non-coding regions have been identified, all of which can lead to decreased or absent enzyme function (Memon et al., 2016). Polymorphisms in the UGT1A1 gene among individuals cause differences in baseline bilirubin levels, which may affect the body’s sensitivity to the toxicity of reactive oxygen species and ultimately influence the risk of developing PD and disease progression (Miners et al., 2002).

Emerging drug delivery strategies aim to overcome these limitations. Firstly, administration of exogenous bilirubin via nanoparticles. Bilirubin-based nanomedicines offer higher solubility, greater delivery efficiency and satisfactory therapeutic outcomes; they enable targeted delivery to the central nervous system, thereby avoiding side effects in non-target tissues and cells (Jagaran and Singh, 2022; Yao et al., 2020a). Secondly, activating the heme redox pathway through BRUP-1 (intracellular bilirubin modulator). BRUP-1 activates the Nrf2-HO-1 axis by directly inhibiting the interaction between Nrf2 and Kelch-like ECH-associated protein 1 (Keap1), and increases the production of BR, thereby providing an approach more in line with physiological mechanisms (Kataura et al., 2020). Thirdly, local drug administration strategies, for example, nasal administration can avoid the risk of systemic toxicity. Lastly, gene therapy targeting the expression of UGT1A1 or HO-1 can achieve continuous and controllable regulation of bilirubin metabolism.

In summary, clinical trial design must take into account the dual role of bilirubin, including formulating a rigorous dose escalation scheme, stratifying patients based on UGT1A1 genotype, and simultaneously monitoring signs of both efficacy and bilirubin neurotoxicity. Recent studies have confirmed that bilirubin can prevent the loss of TH^+^ dopaminergic neurons *in vitro* PD models by acting on TNF-*α*, providing a mechanistic basis for related trials (Jayanti et al., 2022).

## 5 Conclusion

The significant rise in the number of patients with PD has rendered their clinical diagnosis and treatment increasingly challenging. The role of bilirubin in the pathogenesis of PD does not operate in isolation, but rather as part of an interconnected network and it is expected to become a new option for the treatment of PD. In conclusion, BR is emerging as a promising disease-modifying candidate for PD rather than merely a metabolic waste product. Future research should focus on elucidating the precise dose–response window for BR’s neuroprotective effects and developing targeted delivery systems to minimize systemic toxicity.
